# Metrnl and macrophage polarization: role in skeletal muscle homeostasis and therapeutic potential

**DOI:** 10.3389/fimmu.2026.1843626

**Published:** 2026-06-18

**Authors:** Bo Huang, Haihua Xie, Weilu Li, Yimin Chen, Panpan Mu, Lingshan Yu, Yuebing Li, Xuqiu Zeng, Jiadong Zang, Liang Peng

**Affiliations:** 1 College of Acupuncture and Tuina and Rehabilitation, Hunan University of Chinese Medicine, Changsha, China; 2 College of Chinese Medicine, Hunan University of Chinese Medicine, Changsha, China; 3 Department of Science and Technology, Hunan University of Chinese Medicine, Changsha, China

**Keywords:** glucose metabolism, inflammation, macrophage polarization, meteorin-like protein, myokines, sarcopenia, skeletal muscle homeostasis

## Abstract

Macrophage polarization has a significant influence on the immune microenvironment of skeletal muscle, regulating metabolic and repair homeostasis. Meteorin-like protein (Metrnl), a recently identified myokine, is implicated in immunity, metabolism, and tissue remodeling. It regulates macrophage polarization through a complex, integrated signaling network, conferring multiple metabolic benefits on skeletal muscle. This review outlines the dynamics of macrophage polarization in maintaining skeletal muscle homeostasis and discusses the signaling pathways through which Metrnl exerts its effects. Drawing on human and rodent *in vivo* studies, the focus is on the pivotal role of this regulatory axis in skeletal muscle destabilization, particularly in glucose metabolic disorders and age-related sarcopenia. Notably, Metrnl acts as a bidirectional regulator whose biological effects are highly tissue- and disease-dependent. The review further concludes by examining potential pathological mechanisms linking Metrnl-modulated macrophage polarization to skeletal muscle microenvironmental homeostasis, and highlights unresolved questions regarding Metrnl receptor distribution and subset-specific macrophage regulation, putting forward multi-omics and *in vivo* imaging technologies as core avenues for subsequent exploration.

## 1 Introduction

Skeletal muscle homeostasis involves not only the local equilibrium among muscle fibers, surrounding vasculature, nerves, and infiltrating cells within the tissue itself but also systemic crosstalk between muscle and other organs, especially during lipid metabolism and insulin secretion (1, 2). As the largest tissue in the human body, accounting for 40% to 50% of total body mass (3), skeletal muscle is far more than a mere effector of movement and force generation. It serves as the primary protein reservoir and secretes a variety of myokines through autocrine and paracrine signaling (4), thereby playing an extensive role in systemic metabolism, energy homeostasis, and the pathogenesis of chronic diseases. Consequently, skeletal muscle is now widely recognized as an endocrine organ (5). Both extrinsic factors, such as mechanical stress, and intrinsic factors, including gene mutations within myocytes or organismal aging, can lead to the disruption of skeletal muscle homeostasis. When abnormalities in muscle mass and function arise, this breakdown of homeostasis directly compromises key physiological processes, including metabolism, locomotion, and respiration (6, 7). Such disturbances contribute to the development of metabolic disorders and motor impairments, ultimately exerting a profound impact on human health and quality of life.

Within the regulatory network governing skeletal muscle homeostasis, macrophages are well-established key players. Through dynamic phenotypic switching, these cells orchestrate inflammation resolution, muscle repair, and vascular remodeling, acting as central cellular mediators in maintaining skeletal muscle homeostasis (8, 9). Meanwhile, skeletal muscle acts as an endocrine organ by secreting myokines, forming a critical link between muscle and the immune system. Accumulating evidence has identified meteorin-like (Metrnl), a pivotal myokine secreted by skeletal muscle, as modulating macrophage polarization to regulate metabolic homeostasis, inflammation responses, and muscle regeneration (10–12). The unique expression profile and specific role of Metrnl in directing macrophage polarization have established it as an emerging hotspot in skeletal muscle homeostasis research. This review aims to provide a comprehensive overview of the biological characteristics of Metrnl, the regulation of its secretion as a myokine, and its effects on macrophage polarization. We propose that Metrnl serves as a key signaling molecule mediating crosstalk between muscle homeostasis and immune regulation. By driving macrophages toward a pro-reparative phenotype, Metrnl plays a critical role in muscle injury repair, metabolic balance, and tissue microenvironmental stability. Elucidating these mechanisms not only holds significant theoretical value but may also offer novel therapeutic targets for skeletal muscle disorders, metabolic syndrome, and related conditions.

To ensure transparency and minimize selection bias, we conducted a systematic literature search. The search was performed in PubMed, Web of Science, the Cochrane Library, CNKI and Wanfang from inception to February 2026, with no language restrictions. Details of the literature search strategy and PRISMA flowchart are provided in the **Supplementary Materials**.

## 2 Macrophage polarization: a key regulator of skeletal muscle homeostasis

### 2.1 Macrophage phenotypes and functions

**Figure 1:**
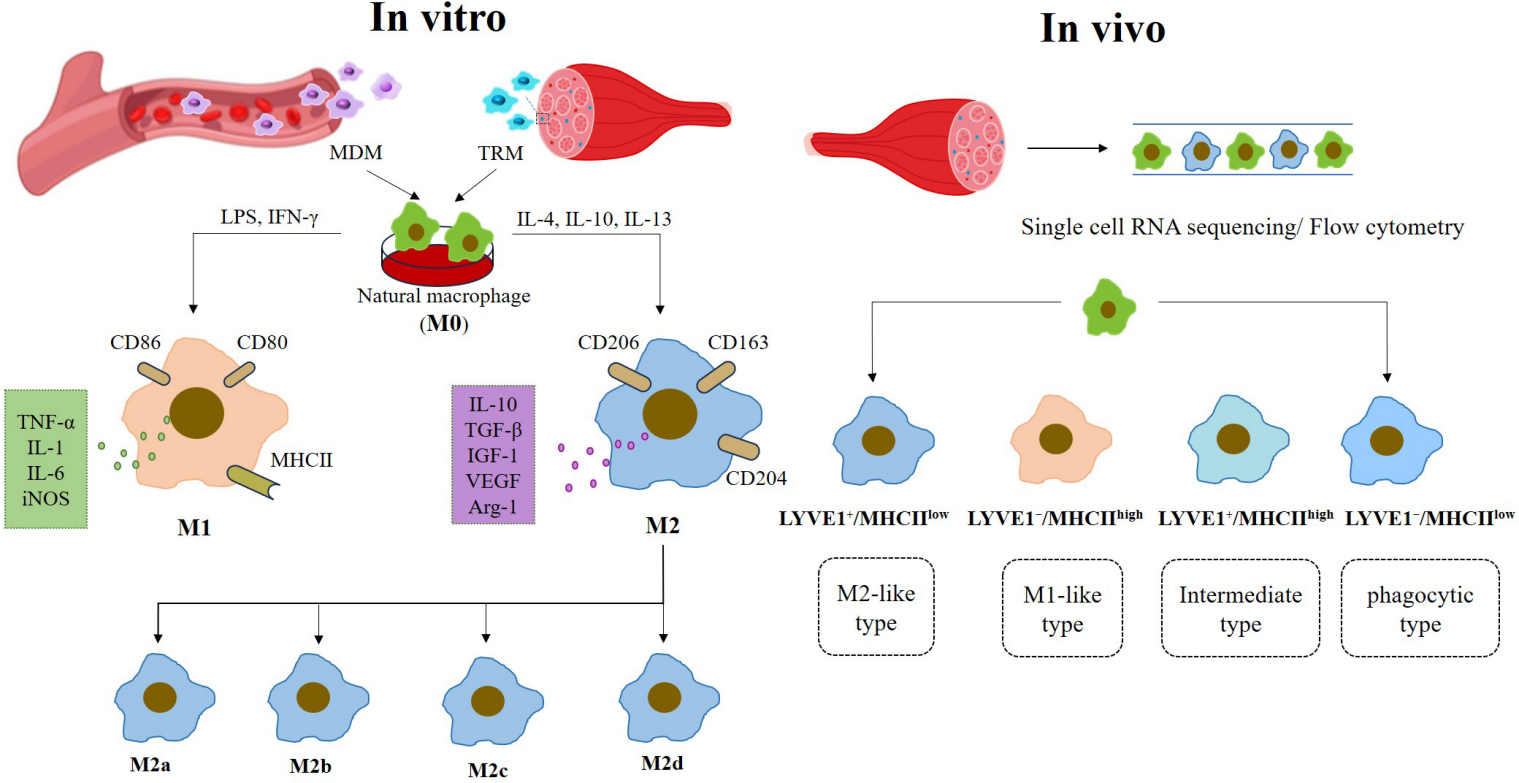
Phenotypes and functions of macrophages in muscle tissue. The pool of M0 macrophages in muscle tissue is collectively constituted by tissue-resident macrophages (TRM), which are long-lived and locally maintained, and monocyte-derived macrophages (MDM), which are recruited from the circulation. *In vitro*, upon activation by pro-inflammatory signals, including LPS and IFN-γ, M0 macrophages undergo a series of phenotypic and functional changes, polarizing into M1 macrophages that secrete an array of proinflammatory factors, such as TNF-α, IL-1, and iNOS, thereby participating in the initiation and progression of inflammatory responses, pathogens elimination, and anti-tumor. Under the regulatory effect of anti-inflammatory cytokines, including IL-4, IL-10, and IL-13, macrophages polarize toward the M2 phenotype. This phenotype can be further subdivided into M2a, M2b, M2c, and M2d subtypes, each distinguished by specific stimulating factors and secreted cytokines. These subtypes are primarily involved in the anti-inflammation, tissue repair and remodeling, angiogenesis, and the regulation of immune homeostasis. *In vivo*, flow cytometry and single-cell transcriptomics have revealed four macrophage subsets—M2-like (LYVE1^+^/MHCII^low^), M1-like (LYVE1^-^/MHCII^high^), an intermediate LYVE1^+^/MHCII^high^ type, and a novel LYVE1^-^/MHCII^low^ population—that collectively drive the dynamic changes in the skeletal muscle tissue microenvironment.

Macrophages within skeletal muscle constitute a heterogeneous cell population. Their polarization is dynamically regulated by an interplay of factors, including cellular origin, microenvironmental cues, and the functional demands of the surrounding tissue. Upon specific signals, quiescent M0 macrophages polarize into either the pro-inflammatory phenotype or the anti-inflammatory/reparative phenotype. This phenotypic transition is central to maintaining skeletal muscle homeostasis, facilitating injury repair, and influencing disease progression (**Figure 1**). Conversely, dysregulated macrophage polarization inevitably disrupts skeletal muscle homeostasis.

#### 2.1.1 Origin and subtypes of muscle macrophages

An essential component of the innate immune system is the macrophage. They are divided into two primary categories based on their cellular origin: tissue-resident macrophages (TRMs) and monocyte-derived macrophages (MDMs). Hematopoietic stem cells in the bone marrow are the source of MDMs. Peripheral blood monocytes are drawn to areas of muscle injury in reaction to mechanical stress, ischemia, infection, or other tissue insults. There, they develop into MDMs (13, 14). The main function of these cells is host defense (15). TRMs, on the other hand, are produced from hematopoietic progenitors in the fetal liver or embryonic yolk sac and sustain their population through *in situ* proliferation after birth (16). They are essential for maintaining skeletal muscle homeostasis and are mostly found in the perimysium, endomysium, and perivascular areas (17, 18).

The diversity in macrophage classification arises from differences in developmental lineage, shaping by the local microenvironment, and plasticity in real−time functional states. This dictates that both the classical M1/M2 dichotomy and novel multi−omics−based subset classifications merely approach a continuous and complex spectrum of cellular identity at different scales.

*In vitro*, macrophages can be simply classified into classical M1 or alternative M2 subtypes. Triggering factors such as interferon−gamma (IFNγ) and lipopolysaccharide (LPS) polarize macrophages toward the M1 state, and polarized M1 macrophages acquire pro−inflammatory, pathogen−clearance, and anti−tumor properties, partially through the inducible nitric oxide synthase (iNOS) pathway. M2 macrophages, depending on the stimulating factors, can be further divided into four subtypes: M2a, M2b, M2c, and M2d.By secreting pro-fibrotic mediators such fibronectin, insulin-like growth factor (IGF), and transforming growth factor-β (TGF-β), M2a macrophages stimulated by Interleukin (IL)-4 or IL-13 aid in tissue healing (19). M2b macrophages have strong immunoregulatory and anti-inflammatory actions and generate large amounts of IL-10 when activated by immune complexes and TLR agonists (20). M2c macrophages, polarized by IL-10, TGF-β, or glucocorticoids, are characterized by high Mer receptor tyrosine kinase expression and robust TGF-β and IL-10 secretion. They mediate clearance of apoptotic cells, suppress immune responses, promote angiogenesis, and contribute to tissue fibrosis (21, 22). Induced by IL-6, TLR agonists, and A2 adenosine receptor agonists, M2d macrophages exhibit a unique cytokine profile with low levels of IL-12, tumor necrosis factor-α (TNF-α), and IL-1β and elevated levels of IL-10, TGF-β, and vascular endothelial growth factor (VEGF), which promotes angiogenesis and cancer metastasis (23).

In the past, macrophages were simply categorized into M1 and M2 phenotypes based on their pro-inflammatory versus anti-inflammatory and reparative functions. However, the extensive application of flow cytometry and single-cell RNA sequencing has proven this simplistic dichotomy insufficient to depict the dynamic functional states of tissue-resident macrophages. In skeletal muscle, for example, many macrophages simultaneously express both M1- and M2-associated surface markers including CD86 and CD206, indicating that traditional markers alone cannot accurately define their functional phenotypes (24).

To identify more practical classification criteria, Krasniewski et al. (25) demonstrated that the membrane protein LYVE1 roughly divides mouse skeletal muscle macrophages into two groups: LYVE1^+^ macrophages exhibit M2-like properties and participate in angiogenesis and tissue repair, whereas LYVE1^-^ macrophages exert pro-inflammation effects and antigen presentation resembling the M1 phenotype. Combined assessment of MHCII expression further subdivides these cells into four subsets: M2-like (LYVE1^+^/MHCII^low^), M1-like (LYVE1^-^/MHCII^high^), an intermediate subset with mixed functional characteristics (LYVE1^+^/MHCII^high^), and a newly identified LYVE1^-^/MHCII^low^ population. Approximately half of cells within this unique subset possess potent phagocytic activity and are defined as hyperphagocytic macrophages. These cells also co-express CD47 to evade phagocytic clearance.

Single-cell RNA-seq has further uncovered multiple functionally specialized macrophage subsets. A Gpnmb^+^Spp1^+^ subset emerges transiently following acute muscle injury yet sustains its presence in chronically damaged tissue. This subset harbors gene signatures characteristic of scar-associated macrophages and contributes to extracellular matrix remodeling and tissue fibrosis (26). Another S100A8^+^/A9^+^ cluster expresses high levels of pro-inflammatory factors and is associated with inflammatory diseases (25). During the early phase of muscle injury, the ADAMTS1^+^ mixed macrophage population inhibits NOTCH1 signaling and facilitates the activation of satellite cells for tissue repair (27). Furthermore, a newly identified Mac1 subset promotes satellite cell proliferation via the HGF/c-Met pathway by inhibiting Cdkn1b expression (28). In dystrophic muscle, most macrophages deviate from classic M1 and M2 profiles; these cells abundantly produce profibrotic factors galectin-3 and osteopontin, strongly associating with fibrotic progression and pathological dystrophic microenvironments (9).

Collectively, the M1/M2 dichotomy merely reflects the extreme polarization states induced *in vitro* and fails to capture the true diversity of macrophages *in vivo*. The multiple functional subsets defined by LYVE1, MHCII, and single-cell transcriptomics clearly demonstrate that skeletal muscle macrophages represent a continuous spectrum shaped by developmental origin, local microenvironment, age, and pathological conditions. Future studies should focus on the dynamic transitions among these subsets and their specific roles in disease.

#### 2.1.2 Functional traits of muscle macrophages

Macrophage polarization is governed by the dynamic interplay of cytokines and chemokines, and is further shaped by signals from other immune cells or the tumor microenvironment, resulting in functionally heterogeneous phenotypes. Classically activated M1 macrophages, generally associated with pro-inflammatory responses, are induced by lipopolysaccharide (LPS) and interferon (IFN)-γ and secrete numerous pro-inflammatory mediators, including TNF-α, IL-1, IL-6, and inducible nitric oxide synthase (iNOS) (29), thereby mediating effective elimination of bacteria, viruses, and malignant cells. Moreover, M1-like macrophages exhibit potent phagocytic activity for clearing pathogens and dead cells (30, 31). Internalized material is enclosed in vesicles that fuse with lysosomes, where degradation occurs via lysosomal hydrolases (32). Accordingly, M1-like macrophages play critical roles in host defense and antitumor immunity. However, their prolonged or excessive activation can drive chronic inflammatory diseases (33), necessitating tight regulatory control to prevent persistent inflammation within the tissue microenvironment. In contrast, M2-like macrophages, primarily involved in tissue repair and inflammation resolution, are induced by anti-inflammatory signals including IL-4, IL-13, and IL-10. M2-like macrophages are characterized by the secretion of IL-10, TGF-β, IGF-1, VEGF, and other growth factors, along with upregulation of Arginase (Arg)-1 and surface markers such as CD163, CD204, and CD206. This molecular profile equips them to suppress inflammation and promote tissue repair (34–37). M2-like macrophages also facilitate tissue debris clearance, wound healing, and angiogenesis (38), thereby preserving tissue homeostasis and immune balance. Phenotypic switching in macrophages is accompanied by metabolic reprogramming. M1-like macrophages primarily rely on high-rate glycolysis for rapid energy generation to fuel pro-inflammatory responses, whereas M2-like macrophages predominantly utilize oxidative phosphorylation or fatty acid oxidation to support their anti-inflammatory and reparative functions (39, 40). Of note, within the tumor microenvironment, tumor-associated macrophages (TAMs) typically exhibit an M2-like phenotype and promote tumor growth and progression (41).

### 2.2 Macrophage polarization regulates skeletal muscle homeostasis

**Figure 2:**
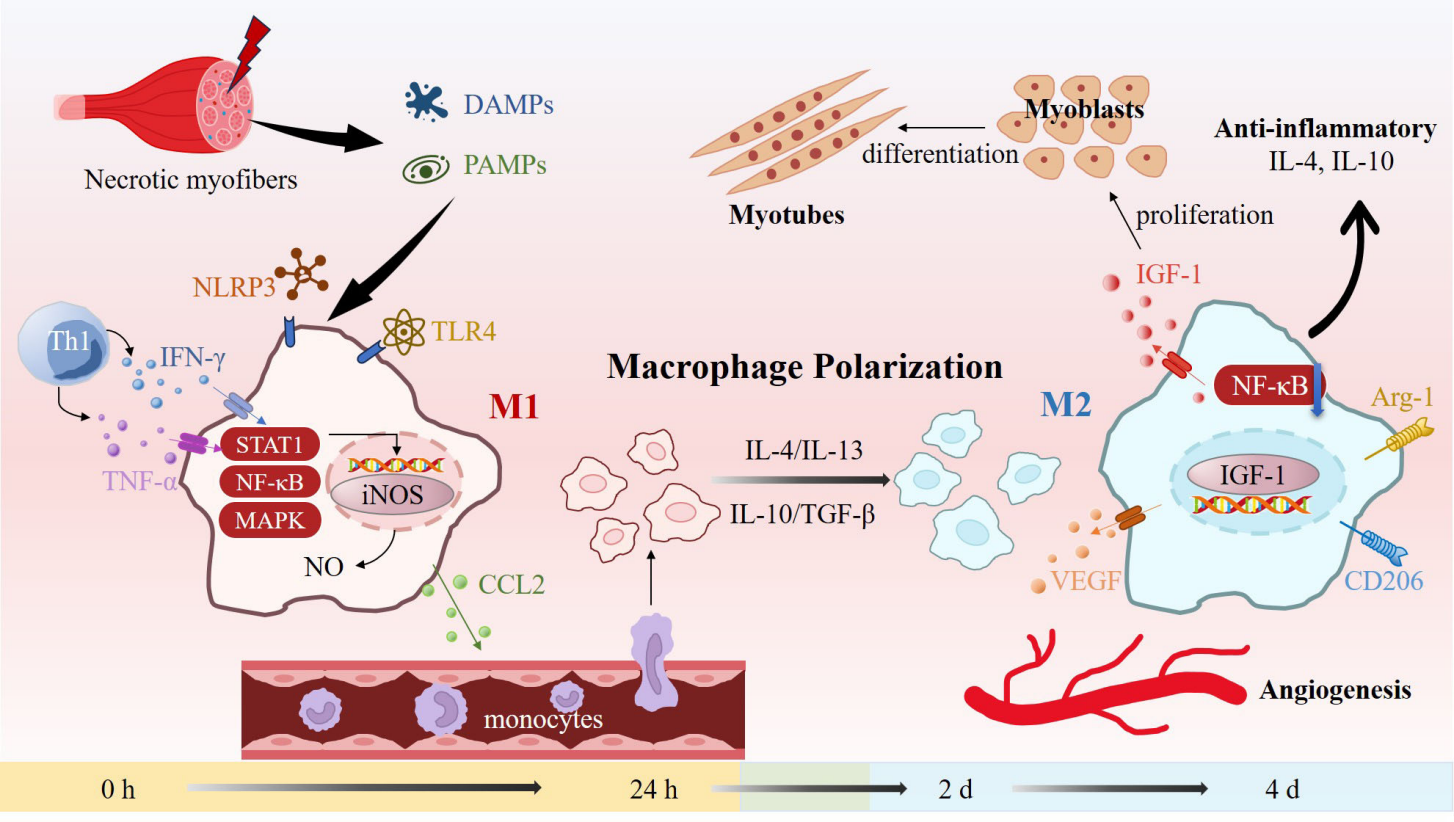
Macrophage polarization in injured skeletal muscle. Following skeletal muscle injury, damage-associated molecular patterns (DAMPs) and pathogen-associated molecular patterns (PAMPs) released by necrotic myofibers induce M1 macrophage polarization, accompanied by activation of STAT1, NF-κB, and MAPK signaling pathways that mediate post-injury inflammatory responses. M1-like macrophages secrete CCL2 to recruit circulating monocytes into the injured site, which further differentiate into M1-like macrophages under local stimulatory factors. As the injury transitions to the repair phase, M1-like macrophages polarize into M2-like macrophages under the regulation of IL-4, IL-10, and TGF-β. M2-like macrophages secrete IGF-1 to promote myocyte proliferation and differentiation, VEGF to facilitate neovascularization, and IL-4/IL-10 to accelerate inflammation resolution.

Skeletal muscle homeostasis hinges on the dynamic equilibrium between M1-like and M2-like macrophage polarization; disturbing this balance directly compromises muscle function (42, 43). During muscle repair, macrophages undergo a sequential polarization program, initiating with a pro-inflammatory phase followed by an anti-inflammatory, reparative phase. This precisely regulated inflammatory-to-reparative transition is essential for efficient muscle regeneration and functional restoration (43) (**Figure 2**).

In the early stage of injury, damage-associated molecular patterns (DAMPs) and pathogen-associated molecular patterns (PAMPs) released by necrotic myofibers activate macrophages through pattern recognition receptors, including toll-like receptor 4 (TLR4) and NOD-like receptor thermal protein domain associated protein 3 (NLRP3) (44, 45). Meanwhile, IFN−γ, TNF−α, and other T-cell-derived cytokines activate the signal transducer and activator of transcription (STAT) -1, nuclear factor kappa-B (NF−κB), and mitogen-activated protein kinase (MAPK) pathways (46–48), driving M1-like polarization. M1-like macrophages generate NO via high iNOS expression and, together with lysosomal enzymes, phagocytose and degrade necrotic myofibers (49). In parallel, TRMs recruit circulating monocytes by secreting Chemokine ligand 2 (CCL2/MCP-1), promoting their differentiation into M1-like macrophages that peak at approximately 24 h post-injury (13). Further single-cell RNA sequencing revealed that a damage-associated subtype of M1-like macrophages, namely the Gpnmb^+^Spp1^+^ macrophage subpopulation, was significantly expanded upon acute injury (26). TRMs also upregulate major histocompatibility complex class II (MHC−II) expression to enhance antigen−presenting capacity (50). However, sustained M1-like activation leads to pathological outcomes. In chronic inflammatory myopathies like polymyositis, persistent TLR4/NF−κB activation drives excessive M1-like macrophage accumulation. TNF−α and IL−1β from M1 macrophages suppress myogenic differentiation 1 (MyoD) expression in satellite cells, inhibiting myofiber regeneration (51). Furthermore, TGF−β1 and platelet-derived growth factor (PDGF) activate fibroblasts, promoting type I collagen deposition and subsequent myotendinous interstitial fibrosis, which impairs muscle contractile function (52, 53).

The M1-to-M2 phenotypic switch peaks 2–4 days post-injury, marking the transition of the muscle microenvironment into an anti-inflammatory and reparative phase (54, 55). During this phase, M2-like macrophages drive myoblast differentiation and myofiber growth through the secretion of IL−4 and IGF−1, while also producing anti-inflammatory cytokines such as IL−10 to promote inflammation resolution (42, 56). Among the M2 subsets, the IL-4/IL-13-induced M2a subtype (57) is characterized by high expression of Arg-1 and CD206, which drive arginine metabolism to produce polyamines that support myofiber regeneration (58). The IL-10/TGF-β-induced M2c subtype, in turn, promotes satellite cell proliferation and differentiation, enhances angiogenesis via IGF-1 and VEGF secretion, and helps terminate inflammation by suppressing NF-κB activity (51, 59). Conversely, insufficient M2-like polarization leads to impaired muscle repair. In aged muscle, reduced TRM numbers and compromised STAT6/PPAR-γ signaling (60) result in defective clearance of necrotic myofibers. Inadequate IGF-1 signaling further impairs satellite cell proliferation and differentiation, while reduced angiogenesis leads to local hypoxia, finally contributed to muscle mass loss and the development of sarcopenia (61). In the setting of diabetes, chronic hyperglycemia suppresses IL-10 secretion by M2-like macrophages, thereby establishing a vicious cycle of sustained polarization imbalance, delayed repair, and progressive homeostatic disruption (62).

Collectively, these findings position the dynamic balance of macrophage polarization as a central mechanism governing skeletal muscle homeostasis, through its precise coordination of the inflammation-to-repair transition, extracellular matrix remodeling, and angiogenesis. Accordingly, disruption of this balance represents a key pathological driver of chronic myopathies, sarcopenia, and metabolic muscle injury.

## 3 Molecular characteristics and expression of Metrnl

Metrnl (also known as IL41, IL-39, and subfatin) is a recently identified secreted protein that shares high amino acid sequence homology with Meteorin, hence its alternative name, Meteorin-like factor (63). It was first described in 2007 in the doctoral thesis of Weiyan Gong, who identified it as an osteoblast-derived secretory protein potentially involved in regulating bone formation (64). Subsequent studies have since uncovered the broad tissue distribution and pleiotropic functions of Metrnl. In 2014, Rao et al. (65) showed that Metrnl is most abundant in mouse skeletal and cardiac muscle, followed by white and brown adipose tissue, with lower expression detected in the kidney, spleen, and lung. They further proposed that Metrnl plays a key role in suppressing adipose tissue inflammation and promoting browning of white adipose tissue. In the same year, Li et al. deposited this protein in the UniProt knowledgebase (66). Using human gene expression databases, Ushach et al. (67) further reported high Metrnl expression in activated monocytes and in mucosal tissues, including those of the skin, oral cavity, esophagus, trachea, and bronchus, with particularly robust levels in the tongue muscle. These observations suggest that Metrnl may be involved in both innate and adaptive immunity, though its precise functions across different tissues remain to be fully defined.

At the molecular level, the human and mouse Metrnl genes share 77.49% sequence homology, indicating strong evolutionary conservation. The human Metrnl gene, located on chromosome 17, encodes a 311-amino acid precursor protein; following cleavage of the N-terminal signal peptide (approximately 45 amino acids), the mature secreted protein consists of 266 amino acids (68). Metrnl contains a glycosylation site, and this post-translational modification is thought to critically influence its stability, secretion, and biological activity (66). Reboll et al. (69) further identified monocyte/macrophage-derived Metrnl as a high-affinity ligand for the stem cell factor receptor KIT. Collectively, Metrnl is a widely expressed factor that plays key roles in nutrient homeostasis, metabolic regulation, and anti-inflammatory processes. Its expression and biological activity are notably context-dependent and tissue-specific (70, 71).

Metrnl expression is tightly regulated by diverse physiological and pathological stimuli (**Table 1**). Among these, exercise stands out as one of the most potent inducers of Metrnl expression. Both acute and short-term high-intensity interval training (HIIT) significantly upregulate Metrnl levels in skeletal muscle (73). Additionally, cold exposure robustly induces Metrnl expression. By promoting the expression of thermogenesis-related genes and interleukins, and by facilitating alternative macrophage activation, Metrnl enhances systemic thermogenesis (65, 74). Beyond these stimuli, a range of inflammatory cytokines, metabolic states, nutritional status, and pathological conditions also influence Metrnl synthesis and secretion. Metrnl production is induced by TNF−α, IL−17A, IL−12, and IL−4, but suppressed by IFN−γ and TGF−β (72). Clinically, circulating Metrnl levels are reduced in overweight or obese individuals (75). During atherosclerosis progression, Metrnl levels decline, but recover during the resolution phase (76). Notably, seemingly contradictory findings have emerged from other clinical studies. Specifically, patients with type 2 diabetes and carotid atherosclerotic plaques show higher circulating Metrnl levels than those without plaques (77). Furthermore, serum Metrnl is elevated in anti-neutrophil cytoplasmic antibody-associated vasculitis (AAV), where it correlates with disease activity and renal impairment (78). A similar association is also observed in patients with myasthenia gravis (MG) and invasive candidiasis (79, 80). In acute infection or sterile injury, Metrnl primarily exerts anti-inflammatory and pro-repair functions. However, when immune dysregulation is chronic or excessive, elevated Metrnl may reflect compensatory feedback that ultimately fails to restore homeostasis, or in some contexts, becomes maladaptive. For instance, in autoimmune diseases, increased Metrnl could participated in abnormal regulation of B-cell subsets and imbalanced differentiation of T-cell (79, 81). In contrast, the association of Metrnl with poor prognosis in invasive candidiasis may be attributed to the distinct inflammatory context. in fungal infections, Metrnl exerts negative effects by inhibiting the phagocytic and killing abilities of macrophages against fungi, thereby promoting fungal invasion of the body (80). Collectively, these findings position Metrnl not merely as a protective or pathogenic factor, but rather as a bidirectional regulator with striking tissue specificity and disease context dependency. Further studies dissecting Metrnl signaling in specific immune subsets are needed to resolve these apparent contradictions and to provide a more solid theoretical basis for its potential application as a therapeutic target.

**Table 1:** Summary of literature on Metrnl regulation, models, and effects.

Authors/year	Model/context	Stimulus/condition	Metrnl expression	Effects
RR.Rao, et al., 2014 (60)	Muscle-specific PGC-1α4 transgenic mice	Exercise	Increased in skeletal muscle	Stimulates energy expenditure, improves glucose tolerance, beige fat thermogenesis and anti-inflammatory responses
		Cold exposure	Increased in serum	
I. Ushach, et al., 2015 (62)	Macrophages	IL-4	Increased	Involves in both innate and acquired immune responses
		IFN-γ/LPS	Decreased	
	Human biopsy sample (psoriasis, atopic dermatitis, prurigo nodularis, actinic keratosis, rheumatoid arthritis)	–	Increased	
M. Eaton, et al., 2018 (67)	Skeletal muscle biopsy samples	HIIT	Increased	–
I. Ushach, et al., 2018 (69)	Bone marrow macrophages	TNF-α, IL-17α, IL-12, and IL-4	Induced	Participates in the control of inflammatory responses
		IFN-γ and TGF-β	Inhibited	
S. M. Tayebi, et al., 2024 (68)	Diabetic male rats	Cold-water swimming + cinnamon extract	Increased in serum	Improve diabetes-related indices
Z. Wang, et al., 2024 (72)	Myasthenia gravis patients	–	Increased in serum	High serum Metrnl is associated with the severity of the myasthenia gravis
X. Wang, et al., 2026 (70)	Obese adults	–	Decreased	Low serum Metrnl is closely associated with early lipid abnormalities and insulin resistance
X. Chen, et al., 2026 (71)	Atherosclerosis model (apoE^−^/^−^ mice)	High-fat, high-cholesterol diet	Decreased	Metrnl suppresses VSMC foam cell formation and atherosclerosis via KIT signaling, reducing ER stress and subsequent cholesterol uptake.
X. Deng, et al., 2026 (73)	Patients with type 2 diabetes mellitus	–	Increased in serum	Elevated Metrnl levels are a risk factor for carotid plaque formation in T2DM patients
Y. Jia, et al., 2026 (74)	Patients with anti-neutrophil cytoplasmic antibody-associated vasculitis	–	Increased in serum	High serum Metrnl is associated with AAV activity and renal dysfunction
J. Liu, et al., 2026 (75)	Patients with candidemia	–	Increased in serum	Higher circulating Metrnl concentration is associated with poor survival

## 4 Metrnl and macrophages

**Figure 3:**
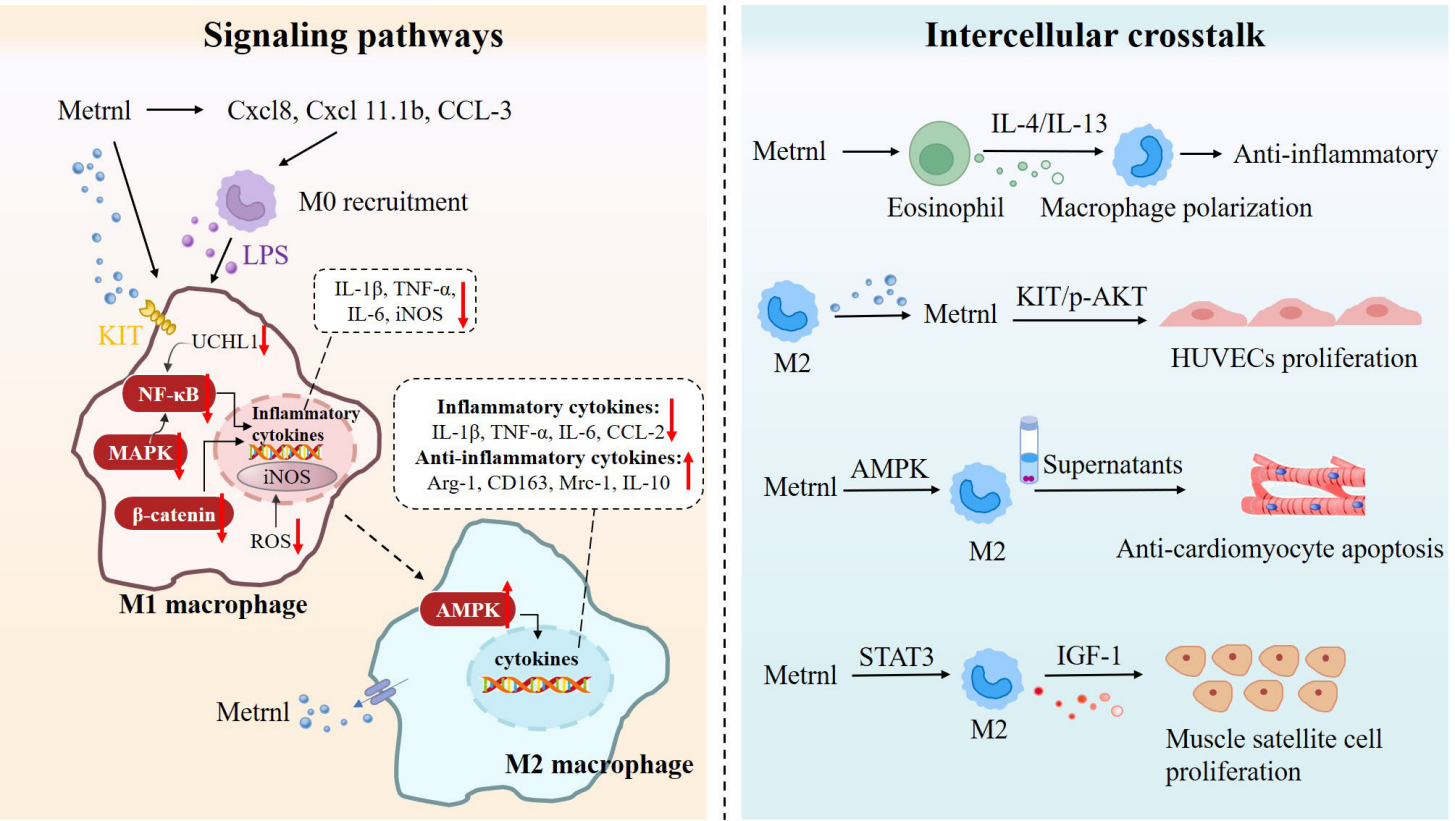
Metrnl regulates macrophages: signaling pathways and cellular crosstalk. Metrnl directly regulates macrophage polarization via multiple signaling pathways. On the one hand, Metrnl inhibits the UCHL1 and MAPK pathways, leading to downregulation of downstream NF-κB signaling; it also suppresses the β-catenin signaling pathway. These effects result in reduced secretion of pro-inflammatory cytokines (IL-1β, TNF-α, IL-6, iNOS) from M1-like macrophages. On the other hand, Metrnl activates the AMPK pathway, promoting M2-like macrophages to produce anti-inflammatory factors such as Arg-1, CD163, Mrc-1, and IL-10, while inhibiting the expression of IL-1β and TNF-α. Additionally, Metrnl induces M2-like macrophages to secrete more Metrnl, which contributes to tissue repair. Furthermore, Metrnl facilitates crosstalk between macrophages and various tissue cells, including eosinophils, vascular endothelial cells, cardiomyocytes, and muscle satellite cells.

As a recently identified myokine, Metrnl exerts a key function by precisely regulating macrophage phenotypic switching, thereby driving inflammatory microenvironment remodeling and promoting tissue homeostasis. This process involves the activation of multiple signaling pathways and extensive intercellular crosstalk (**Figure 3**), and ultimately drives inflammation resolution, facilitates tissue repair, and improves metabolic disorders.

### 4.1 Metrnl directly regulates macrophages via signaling pathways

Metrnl exerts bidirectional regulation on macrophage polarization through specific receptors and downstream signaling cascades. It not only recruits macrophages but also suppresses pro-inflammatory M1 polarization while promoting anti-inflammatory, reparative M2 polarization. Ji et al. (82) reported that in pelagic fish, Metrnl upregulates Cxcl8 and Cxcl11.1b mRNA levels, thereby promoting the recruitment, proliferation, and phagocytic activity of primary monocytes/macrophages (Mo/Mφ). Additionally, recombinant Metrnl increases CCL3 levels, which helps restore peritoneal macrophage recruitment and Treg/Th17 balance in Metrnl-deficient mice subjected to sepsis (83).

M1 macrophages are pro-inflammatory, and accumulating evidence indicates that Metrnl suppresses excessive inflammatory cytokine secretion from these cells. In patients with oral squamous cell carcinoma exhibiting high lnc-Metrnl expression, the estimated infiltration levels of four immune cell types, including M1 macrophages, are significantly reduced (84). Chen et al. (85) reported that altered serum Metrnl levels in patients with lupus nephritis are associated with macrophage-mediated inflammatory bias. Compared to healthy controls, serum Metrnl levels in these patients are decreased, and this reduction negatively correlates with TNF-α levels while positively correlating with IL-10 and Arg-1 levels (85). Preclinical studies have shown that in LPS-activated macrophages, Metrnl inhibits the UCHL1/NF-κB pathway by suppressing UCHL1 deubiquitinating activity, leading to sustained NF-κB ubiquitination and degradation. This ultimately downregulates macrophage secretion of pro-inflammatory cytokines such as IL-1β, TNF-α, and IL-6, thereby alleviating the inflammatory microenvironment (86). Furthermore, Metrnl has been shown to downregulate ROS levels in M1-like macrophages, inhibit iNOS^+^ macrophage differentiation via ROS signaling, and enhance macrophage phagocytosis, thereby promoting E. coli clearance (83). In patients with fulminant hepatitis, elevated serum Metrnl is primarily derived from liver macrophages. Du et al. (87) further demonstrated *in vitro* that Metrnl binds specifically to KIT on the macrophage membrane, inhibits MAPK and NF-κB signaling, and reduces TNF and IL-1β secretion, thereby exerting anti-inflammatory effects. In a mouse model of atopic dermatitis, Metrnl binds to KIT, inhibits immune cell expansion, and downregulates inflammatory gene expression by modulating β-catenin levels in the Wnt pathway, thereby alleviating allergic inflammation (11).

Metrnl also promotes selective M2 macrophage activation, which supports anti-inflammatory responses and tissue repair. In AAV9-Metrnβ-injected Metrnβ−/− hearts, expression of the pro-inflammatory factors CCL2 and TNF-α is significantly reduced, whereas the anti-inflammatory factors Arg-1, CD163, and mannose receptor C-1 (Mrc-1) are markedly upregulated. Using the iNOS/Arg ratio as a proxy, Rupérez et al. estimated that the M1/M2 macrophage ratio is significantly reduced in hearts overexpressing Metrnl, while total macrophage numbers remain unchanged. This suggests that Metrnl promotes a non-inflammatory myocardial environment (88). In a mouse model of cigarette smoke-induced lung inflammation, Cen et al. (89) demonstrated that Metrnl pretreatment promotes M2 polarization, leading to increased IL-10 and reduced TNF-α, IL-6, and IL-1β in serum and lung tissue, thereby attenuating pulmonary inflammation. Additionally, Chen et al. demonstrated that Metrnl promotes the polarization of macrophages from M1 to M2 by activating AMPK, thereby reducing inflammatory responses and cardiomyocyte apoptosis, and alleviating myocardial ischemia-reperfusion injury in mice (10).

However, the interaction between Metrnl and macrophages is not always beneficial to the host. Studies have shown that Metrnl is associated with poor prognosis in invasive candidiasis (80). Upon binding to KIT, Metrnl downregulates dectin-1 secretion from macrophages via STAT3 signaling, impairing their phagocytic and fungicidal activity. This negatively regulates anti-fungal immunity in mice and increases disease mortality. These findings indicate that the role of Metrnl in regulating macrophages is complex and context-dependent.

### 4.2 Metrnl regulates macrophages via intercellular crosstalk

The regulation of macrophage polarization by Metrnl also involves intercellular crosstalk, linking skeletal muscle to the immunometabolic environment, cardiovascular diseases, and tissue repair. In adipose tissue, eosinophils are involved in the regulatory effect of Metrnl on macrophages. Metrnl produced by skeletal muscle in response to exercise is released into the bloodstream; circulating Metrnl then stimulates eosinophils to secrete IL-4/IL-13, which induces the alternative activation of macrophages in adipose tissue. This process leads to increased catecholamines and anti-inflammatory factors, resulting in adipose tissue browning, enhanced thermogenesis, elevated energy expenditure, improved glucose tolerance, and attenuated inflammatory responses (65). Furthermore, Metrnl is closely associated with wound healing, which involves crosstalk between macrophages and endothelial cells. Zhao et al. (90) developed a recombinant human Metrnl hydrogel formulation for local administration, which promotes angiogenesis and accelerates wound healing. Song et al. demonstrated that the cytokine Metrnl, produced by keratinocytes and macrophages, activates AKT phosphorylation (S473) in a c-Kit-dependent manner, induces the proliferation of HUVECs, and enhances post-injury angiogenesis and re-epithelialization (91). The regulation of macrophages by Metrnl also impacts cardiomyocytes. Studies in mice with myocardial ischemia/reperfusion injury have shown that Metrnl alleviates inflammatory responses by activating AMPK-mediated M2 macrophage polarization. *In vitro*, supernatants from macrophages treated with Metrnl protect AC16 cardiomyocytes from apoptosis under hypoxia/reoxygenation (H/R) conditions (10). Following skeletal muscle injury, Metrnl released by myofibers not only acts directly on macrophages but also exerts indirect tissue repair effects through crosstalk between macrophages and muscle satellite cells. In skeletal muscle tissue, the majority of Metrnl expression originates from MDMs (92). Metrnl can stimulate macrophage polarization by upregulating STAT3, leading to decreased secretion of pro-inflammatory cytokines such as IL-1β and TNF-α, increased production of anti-inflammatory factors such as Arg-1, and enhanced secretion of IGF-1 by M2-like macrophages. This induces the differentiation of muscle satellite cells, resulting in the massive generation of new myofibers and ultimately promoting the skeletal muscle repair process (12). Additionally, under the influence of Metrnl, macrophages produce more TNF to induce the apoptosis of FAPs, counteracting profibrotic programs and ultimately facilitating muscle recovery in aged mice (93).

Overall, the function of Metrnl in regulating macrophage polarization holds significant value across multiple contexts. Currently, targeting Metrnl to modulate macrophage polarization has emerged as a potential strategy for the treatment of skeletal muscle injury and metabolism-related myopathies; however, further mechanistic investigations are needed to clarify the regulatory differences of Metrnl in distinct pathological microenvironments.

## 5 Regulatory role of the Metrnl-macrophage polarization axis in skeletal muscle homeostasis

Preclinical experiments have established Metrnl as a critical regulator of skeletal muscle homeostasis, with its interaction with macrophage polarization forming a pivotal signaling axis. This regulatory mechanism is orchestrated through macrophage-mediated polarizing pathways that dynamically modulate skeletal muscle steady-state conditions. Specifically, Metrnl exerts its effects by directionally controlling the M1/M2 macrophage polarization switch, thereby playing a specific regulatory role in skeletal muscle glucose-lipid metabolism regulation, injury-induced regeneration and repair, and age-related functional decline. This mechanistic insight not only advances our understanding of skeletal muscle disease pathogenesis but also opens new avenues for targeted therapeutic interventions, offering a fresh perspective on precision medicine approaches for muscular disorders.

### 5.1 Glycolipid metabolism regulation

Skeletal muscle is the primary site of insulin-mediated glucose uptake. When glucose utilization is impaired and insulin resistance develops, the balance between protein synthesis and degradation in skeletal muscle is disrupted (94). Under metabolic stress conditions such as obesity or a high-sugar diet, lipid accumulation and oxidative stress in skeletal muscle drive macrophage polarization toward the pro-inflammatory M1 phenotype. These M1 macrophages secrete large amounts of pro-inflammatory factors such as TNF-α, IL-1β, and IL-6, ultimately disrupting systemic metabolic homeostasis. Consequently, metabolic disorders such as obesity and type 2 diabetes (T2DM) are associated with musculoskeletal complications, and dysregulation of Metrnl may contribute to the development of these obesity- and T2DM-related bone and muscle pathologies (95).

Preclinical studies have established a clear mechanistic link: Metrnl serves as a key regulatory hub by modulating macrophage polarization, thereby reshaping the local immune microenvironment and maintaining glucose and lipid homeostasis in skeletal muscle. Exercise intervention significantly upregulates Metrnl expression in the skeletal muscles of obese mice, inhibiting NLRP3 inflammasome activation through the ERK and p38 MAPK, thereby preventing IL-1β secretion from M1-like macrophages and reducing adiponectin levels in adipocytes. These establishes muscle - adipose tissue crosstalk, ultimately attenuating weight gain, adipose accumulation, and insulin resistance (96). Peroxisome proliferator-activated receptor γ coactivator 1-α (PGC-1α), a master regulator of mitochondrial biogenesis and energy metabolism, upregulates Metrnl expression in skeletal muscle (97, 98).Guo et al. (99) implanted NLS-PGC-1α4 into aged mice and observed significant increases in grip strength, muscle weight, and fiber size. Subsequent RNA-seq analysis identified Metrnl as a potential downstream target of PGC-1α4. Mechanistically, Metrnl replacement promoted adipose tissue macrophages to secrete IGF-1, which subsequently induced muscle hypertrophy via the IGF-1-AKT-mTOR signaling pathway. In addition, Metrnl upregulated glucose transporter 4 (GLUT4) expression and enhanced insulin signaling, thereby promoting the browning of white adipose tissue. These effects contribute to improved systemic energy homeostasis.

The AMPK signaling pathway plays a central role in this process. As a master regulator of cellular energy homeostasis, AMPK dysfunction is a key driver of insulin resistance and metabolic syndrome in both humans and experimental models (100). Metrnl markedly enhances AMPK phosphorylation, thereby exerting metabolic and anti-inflammatory effects. First, Metrnl upregulates mRNA expression of carnitine palmitoyltransferase1 (CPT1), Acyl-CoA oxidase (ACO), and fatty acid binding protein 3 (FABP3) in skeletal muscle via AMPK, thereby promoting fatty acid oxidation (101). Second, Metrnl activates AMPK in skeletal muscle, directly inhibiting NF-κB nuclear translocation and reducing secretion of pro-inflammatory cytokines such as TNF-α, IL-6, and MCP-1, thereby alleviating skeletal muscle inflammation (101). Notably, Metrnl promotes AMPKα2-dependent phosphorylation of histone deacetylase 5 (HDAC5), sequestering it in the cytoplasm and preventing nuclear entry. This relieves HDAC5-mediated repression of GLUT4 transcription, thereby enhancing glucose uptake in skeletal muscle cells (102). This series of regulatory effects ultimately improves systemic glucose metabolism and maintains skeletal muscle homeostasis.

Multiple studies have demonstrated a positive correlation between Metrnl levels and metabolic health. Regular exercise increases skeletal muscle levels of AMPK, PGC-1α, and Metrnl, which in turn promote white adipose tissue browning, reduce lipid accumulation, and improve insulin sensitivity (97). In humans, serum Metrnl levels are reduced in elderly patients with T2DM and sarcopenia, and logistic regression analysis has identified higher Metrnl levels as an independent protective factor against sarcopenia in this population (103). Additionally, Metrnl may mediate post-bariatric surgery improvements in glucose metabolism and muscle mass, processes closely linked to macrophage polarization and systemic metabolic status (104).

In summary, elevated Metrnl levels in tissues or circulation shape an anti-inflammatory skeletal muscle microenvironment via macrophage polarization, enhance insulin sensitivity and glucose metabolism through AMPK/PGC-1α signaling, and ultimately preserve skeletal muscle homeostasis. Notably, most underlying mechanistic insights are established based on animal models, and substantial interspecies differences still hinder direct clinical extrapolation. Collectively, current preclinical and limited clinical data suggest that Metrnl holds tentative therapeutic potential for metabolic disorders such as obesity, T2DM and sarcopenia, as well as relevant musculoskeletal complications. Further large-scale human validation and translational researches are still urgently required to confirm its clinical applicability and definite therapeutic value.

### 5.2 Muscle regeneration and anti-aging regulation

Skeletal muscle possesses remarkable regenerative capacity, and repair following injury depends on the activation, proliferation, and differentiation of muscle satellite cells, as well as myofiber remodeling. As key immune cells in the injury microenvironment, macrophages orchestrate regeneration through the precise temporal dynamics of their polarization. By modulating macrophage polarization, Metrnl provides precise inflammatory control and temporal cues for skeletal muscle regeneration, positioning it as a key regulator of muscle repair.

Metrnl acts directly on M1-like macrophages by activating STAT3 signaling, inducing nuclear translocation of phosphorylated STAT3 (pSTAT3), which then binds to the promoter regions of M2 markers such as IL-10 and Arg-1, driving their expression (12). Meanwhile, Metrnl reshapes the cytokine microenvironment in the injured area, forming a positive feedback loop for macrophage polarization switching. IL-4 and IL-17α regulate Metrnl expression in macrophages, and Metrnl in turn modulates the secretion of cytokines (IL-6, IL-10) and chemokines (CXCL1, CCL2), thereby contributing to the immune-inflammatory response (72). Bath et al. further demonstrated that Metrnl activates Stat3 in macrophages, driving their differentiation toward an anti-inflammatory phenotype (12). This, in turn, induces M2-like macrophages to produce IGF-1, which directly stimulates primary muscle satellite cell proliferation, thereby establishing an inflammation-repair cascade during muscle regeneration (12).

Age-related muscle decline, including sarcopenia, is closely linked to chronic low-grade inflammation, characterized by an increased M1-like macrophage proportion and sustained pro-inflammatory factor expression in skeletal muscle, ultimately driving myofiber damage and metabolic dysfunction (105, 106). With advancing age, muscle mass and function in humans continuously decline, accompanied by a significant reduction in tissue Metrnl levels (93). This phenomenon is recapitulated in animal models: Kuswanto et al. reported that impaired innate immune signaling drives age-related degeneration in mice, suggesting that Metrnl deficiency may underlie age-associated macrophage dysfunction (92). Given its ability to improve muscle physiology and systemic energy balance during aging, Metrnl represents a promising therapeutic target for osteoporosis and age-related metabolic disorders. Lee et al. (93) showed that recombinant Metrnl reverses muscle decline in aged mice. During the initial stage of regeneration, Metrnl induces FAPs apoptosis via TNF-α secreted by M2-like macrophages, reducing myofibrosis and ameliorating age-related muscle dysfunction.

Preclinical studies have firmly established that Metrnl plays a critical protective role in skeletal muscle repair by regulating macrophage polarization and promoting IL-10 and IGF-1 secretion via STAT3 signaling (12). However, recent studies have shown that serum Metrnl levels are significantly elevated in patients with MG (79). These seemingly contradictory findings may actually reflect a compensatory immune response. Metrnl primarily acts as an anti-inflammatory cytokine under inflammatory conditions and may exert a protective role in autoimmune diseases, including MG. Therefore, elevated serum Metrnl levels in MG patients could represent a feedback upregulation aimed at counteracting pathological immune damage, rather than a direct pathogenic factor. Further studies are needed to clarify the functional dynamics of Metrnl at different stages of MG.

## 6 Summary and outlook

In summary, skeletal muscle homeostasis is critically dependent on the dynamic process of macrophage polarization. As a key myokine, Metrnl serves as a critical link between immune cells and skeletal muscle. It regulates macrophage polarization through multiple signaling pathways and modulates crosstalk between macrophages and other cell types, including satellite cells, fibroblasts, and endothelial cells, thereby suppressing inflammation, regulating glucose metabolism, and promoting tissue repair. Given its central role in skeletal muscle homeostasis, Metrnl expression levels closely correlate with aberrant glycolipid metabolism and age-related sarcopenia, positioning it as a key target for understanding both physiological and pathological mechanisms in muscle.

From a clinical perspective, incorporating Metrnl into the molecular diagnostic framework for metabolic myopathies, such as sarcopenia or diabetic myopathy, could aid in assessing disease severity, monitoring progression, and guiding treatment decisions. Furthermore, combining Metrnl-based therapies with established rehabilitation strategies, nutritional interventions, or other myokine-targeted approaches holds significant promise. However, several key questions remain to be addressed. With the ongoing identification of novel macrophage subsets, several questions arise: Are specific subsets linked to differential responses to targeted or immunotherapies? Does Metrnl exert distinct regulatory effects on these populations? KIT is well-established as a specific receptor for Metrnl in macrophages and endothelial cells. Recent evidence further reveals that cardiac smooth muscle cells also express KIT, which mediates the cardioprotective effects of Metrnl (76). Nevertheless, it remains unclear whether skeletal muscle cells express KIT or other distinct receptors that enable Metrnl to directly act on these cells, rather than exerting its regulatory effects on muscle homeostasis via macrophages. The precise mechanisms, signaling pathways, and receptors through which Metrnl regulates macrophage polarization and maintains skeletal muscle homeostasis also require further clarification. Priority should be given to identifying the Metrnl receptor in skeletal muscle. Advanced technologies, including single-cell sequencing, *in vivo* imaging, and proteomics, will be instrumental in addressing these questions. Moreover, research on Metrnl in immune-mediated myopathies, such as polymyositis, and metabolic myopathies remains limited. Further studies are urgently needed to elucidate how Metrnl and macrophages coordinate anti-inflammatory and pro-regenerative responses in skeletal muscle. A deeper understanding of how Metrnl remodels the “difficult-to-repair” pathological microenvironment into a regeneration-permissive niche will provide a theoretical foundation and novel therapeutic strategies for preventing and treating related diseases.
