# Supplementary material for: Metrnl and macrophage polarization: role in skeletal muscle homeostasis and therapeutic potential
Source: Front Immunol. 2026 Jun 18;17:1843626. doi: 10.3389/fimmu.2026.1843626 (PMC13322843; doi:10.3389/fimmu.2026.1843626)

1 Search strategy

PubMed: ("Metrnl"[All Fields] OR ("meteorin-like"[All Fields] AND ("protein s"[All Fields] OR "proteinous"[All Fields] OR "proteins"[Supplementary Concept] OR "proteins"[All Fields] OR "protein"[All Fields] OR "proteins"[MeSH Terms])) OR "subfatin"[All Fields] OR "IL-39"[All Fields] OR "IL41"[All Fields]) AND ("macrophage activation"[MeSH Terms] OR ("macrophage"[All Fields] AND "activation"[All Fields]) OR "macrophage activation"[All Fields] OR ("macrophage"[All Fields] AND "polarization"[All Fields]) OR "macrophage polarization"[All Fields] OR ("macrophagal"[All Fields] OR "macrophage s"[All Fields] OR "macrophageal"[All Fields] OR "macrophages"[MeSH Terms] OR "macrophages"[All Fields] OR "macrophage"[All Fields] OR "macrophagic"[All Fields])) AND ("muscle s"[All Fields] OR "muscles"[MeSH Terms] OR "muscles"[All Fields] OR "muscle"[All Fields] OR ("muscle, skeletal"[MeSH Terms] OR ("muscle"[All Fields] AND "skeletal"[All Fields]) OR "skeletal muscle"[All Fields] OR ("skeletal"[All Fields] AND "muscle"[All Fields])))

Web of Science: (((((((TS = Metrnl) OR (TS = meteorin-like protein)) OR (TS = subfatin)) OR (TS = il-39)) OR (TS = IL41)) AND ((TS = macrophage polarization) OR (TS = macrophage))); ((((((TS = Metrnl) OR (TS = meteorin-like protein)) OR (TS = subfatin)) OR (TS = il-39)) OR (TS = IL41)) AND ((TS = muscle) OR (TS = skeletal muscle)))

The Cochrane Library: (Metrnl or meteorin-like protein or subfatin or IL-39 or IL41) and (macrophage polarization or macrophage) in Title Abstract Keyword; (Metrnl or meteorin-like protein or subfatin or IL-39 or IL41) and (muscle or skeletal muscle) in Title Abstract Keyword

CNKI: ("metrnl" + "镍纹蛋白样蛋白" + "IL41"+ "Subfatin" + "IL39") * ("巨噬细胞" + "巨噬细胞极化"); ("metrnl" + "镍纹蛋白样蛋白" + "IL41"+ "Subfatin" + "IL39") * ("肌肉" + "骨骼肌")

Wanfang: ("镍纹蛋白样蛋白" OR "Metrnl" OR "Subfatin" OR "IL41" OR "IL39") AND (“巨噬细胞” OR “巨噬细胞极化”) ; ("镍纹蛋白样蛋白" OR "Metrnl" OR "Subfatin" OR "IL41" OR "IL39") AND ("肌肉" OR "骨骼肌")

2 PRISMA flowchart


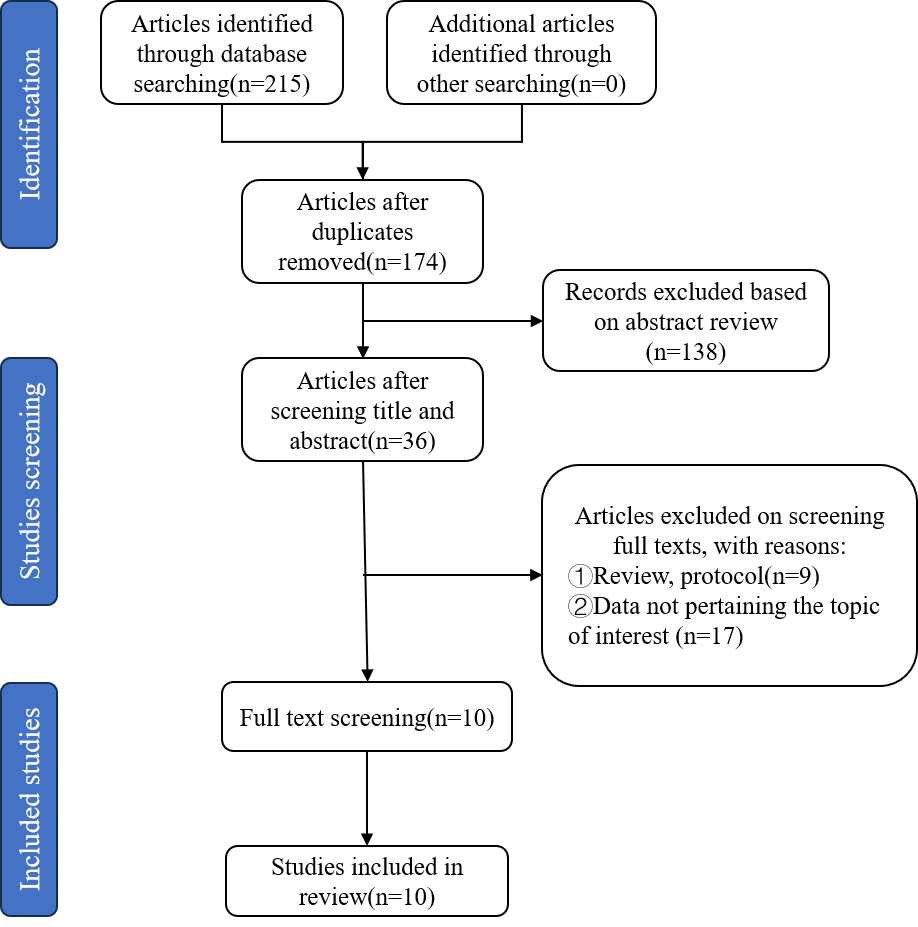

Supplement: Supplementary file 1 [file DataSheet1.docx]
